# Risk communication and informed consent in the medical tourism industry: A thematic content analysis of canadian broker websites

**DOI:** 10.1186/1472-6939-12-17

**Published:** 2011-09-26

**Authors:** Kali Penney, Jeremy Snyder, Valorie A Crooks, Rory Johnston

**Affiliations:** 1 Faculty of Medicine, University of Calgary, 2500 University Drive, NW, Calgary, Alberta, Canada; 2 Faculty of Health Sciences, Simon Fraser University, 8888 University Drive, Burnaby, British Columbia, Canada; 3 Department of Geography, Simon Fraser University, 8888 University Drive, Burnaby, British Columbia, Canada

## Abstract

**Background:**

Medical tourism, thought of as patients seeking non-emergency medical care outside of their home countries, is a growing industry worldwide. Canadians are amongst those engaging in medical tourism, and many are helped in the process of accessing care abroad by medical tourism brokers - agents who specialize in making international medical care arrangements for patients. As a key source of information for these patients, brokers are likely to play an important role in communicating the risks and benefits of undergoing surgery or other procedures abroad to their clientele. This raises important ethical concerns regarding processes such as informed consent and the liability of brokers in the event that complications arise from procedures. The purpose of this article is to examine the language, information, and online marketing of Canadian medical tourism brokers' websites in light of such ethical concerns.

**Methods:**

An exhaustive online search using multiple search engines and keywords was performed to compile a comprehensive directory of English-language Canadian medical tourism brokerage websites. These websites were examined using thematic content analysis, which included identifying informational themes, generating frequency counts of these themes, and comparing trends in these counts to the established literature.

**Results:**

Seventeen websites were identified for inclusion in this study. It was found that Canadian medical tourism broker websites varied widely in scope, content, professionalism and depth of information. Three themes emerged from the thematic content analysis: training and accreditation, risk communication, and business dimensions. Third party accreditation bodies of debatable regulatory value were regularly mentioned on the reviewed websites, and discussion of surgical risk was absent on 47% of the websites reviewed, with limited discussion of risk on the remaining ones. Terminology describing brokers' roles was somewhat inconsistent across the websites. Finally, brokers' roles in follow up care, their prices, and the speed of surgery were the most commonly included business dimensions on the reviewed websites.

**Conclusion:**

Canadian medical tourism brokers currently lack a common standard of care and accreditation, and are widely lacking in providing adequate risk communication for potential medical tourists. This has implications for the informed consent and consequent safety of Canadian medical tourists.

## Background

Medical tourism is a practice whereby patients travel outside of their home country in order to receive medical care. The medical tourism industry is perceived as rapidly expanding, though precise numbers on patient flows are unknown [1]. For the purposes of this paper, we understand medical tourism to entail patients traveling internationally outside of established cross-border care arrangements with accessing medical care as their primary goal, which is usually paid for out of pocket [1]. Patients may decide to pursue medical treatment abroad for any number of reasons, including a desire to gain access to treatments that are not approved or are otherwise available at home, the cost of treatment, and the length of treatment wait times [2]. With smaller, elective procedures it may be the draw of the destination itself or the anonymity provided by going abroad that motivates patient travel [3].

Recently, there have been widely publicized reports in the popular press about medical tourists dying or suffering complications from their surgeries [4-6]. In late 2010, Mahir Mostic, a 35 year old man from Ontario, Canada traveled to Costa Rica to receive the controversial 'liberation therapy' for multiple sclerosis and suffered complications from the surgery. He returned to Costa Rica for follow up care but subsequently died [4]. A woman from Alberta, Canada traveled abroad for breast augmentation in 2010, and returned home with a serious infection that resulted in the eventual loss of the majority of her breast tissue [5]. Cases like these illustrate the possible negative outcomes faced by medical tourists. Any surgery carries with it a certain degree of risk; there is always the possibility of anaesthesia complications, blood clots, uncontrolled bleeding and human error during surgery as well as delayed healing and infection after surgery [1]. Medical tourism, however, carries with it additional risks, including communication gaps between the physician and patient, taking lengthy flights post-operatively, exposure to medical malpractice abroad, difficulties obtaining follow up care, and the danger of infectious disease transmission, among others [1].

A highly visible and influential resource available to patients thinking about going abroad for medical care is the medical tourism broker. For the purpose of this paper a broker is defined as a third party who connects patients to hospitals or doctors in another country. Brokers play a unique role in the practice of medical tourism, and yet remain largely unregulated and poorly understood. The exact percentage of medical tourists who choose to use these brokers' services is unknown, but it is thought that they are likely playing a significant role in medical tourists' health decision-making [7]. For many medical tourists, a broker provides critical information and services that would be difficult to acquire independently, particularly when traveling to a foreign country for the first time. Brokers can help make travel arrangements, suggest physicians and facilities abroad, book surgeries, assist in the transportation and translation of medical records, and help arrange for follow up care and oversee postoperative complications [8,9].

The internet has made it possible for more people than ever before to explore medical treatment options abroad. The vast majority of brokers have a strong online presence and their websites are an important tool for patients seeking information about possible treatments and destinations [10] as well as financing and insurance information [11]. These websites are often the first source of information that patients are exposed to [1,7,11,12]. Therefore, the information about the benefits and risks of engaging in medical tourism presented on these sites may have a strong impact on whether or not and how the prospective medical tourist pursues treatment abroad [12]. While medical tourists working with a broker will typically be given additional information beyond what is available online [6], brokers' websites likely help form the patient's first impression of medical tourism.

Given the risks associated with surgery in general and medical tourism in particular, it is imperative from the standpoint of medical ethics to assess how patients understand these risks and how they are communicated to them by others, especially when coupled with the increasing visibility of medical tourism as a global health services practice. Informed consent is a central ethical value in medical practice [13]. What currently remains unknown is whether medical tourism broker's websites serve to enhance or undermine the capacity of potential medical tourists to make informed choices about engaging in this practice. While potential medical tourists can avail themselves of other sources of information [1,7], the central role of broker websites in forming initial impressions of the safety of medical tourism means that any failure of these sites to communicate risks effectively would raise serious ethical concerns [14]. In this article we examine Canadian medical tourism brokers' involvement in risk communication to prospective patients via their websites in order to address this knowledge gap. We accomplish this through conducting a thematic content analysis of 17 English-language brokerage websites. We seek specifically to analyze the way brokers communicate risk via their promotional websites and the potential impact of their websites on informed consent for patients, which contributes to the nascent literature examining brokers' roles and responsibilities in medical tourism [6,7,11,12,14].

### Risk and Informed Consent in Medical Tourism

Effective communication is essential to medical practice, and medical ethicists emphasize the importance of informed consent as a dimension of effective communication [15]. Informed consent carries with it two levels of moral responsibility on the part of the health professional: adequate information must be communicated to the patient and the patient must understand the information they receive in order to make an informed decision regarding treatment [13]. This is particularly important when considering a medical procedure that is inherently risky or poorly understood [16]. Because the possibility of a negative treatment outcome is appealing neither to the physician nor the patient, there is a tendency to avoid direct discussion of risk, which can lead to patients making decisions without having truly considered the risks involved [17]. Patient assessment of risk is also subject to various biases that can undermine the clear communication of these risks, making it important to factor these biases into risk communication strategies [18].

Medical tourism expands the range of individuals giving patients health advice with the addition of MT brokers and foreign care providers [1], thereby further complicating the communication of risk to these patients and thus ultimately compromising their ability to achieve informed consent. Information about the quality of care available abroad and the possible risks involved in a procedure is important for the patient to have and to understand [1]. While under normal circumstances patients may receive this information from their family doctor or from a nurse or surgeon at a hospital in their home country [18], medical tourists may need to rely on additional sources of information [1,19]. As medical tourism involves travel to a foreign country that patients and their families may be unfamiliar with, this practice creates uncertainties not found in traditional treatment that may add additional risks and informational burdens [20]. Moreover, the choice to travel abroad may strain relationships with the patient's family doctor and other local medical professionals, further cutting off normal means of gaining medical advice [6].

Not only may risk communication be particularly complicated in the case of medical tourism, but the nature of the risks that patients may be exposed to can also be heightened. Long air travel has been shown to increase a person's risk of deep-vein thrombosis (DVT) [21] and it is suspected that this risk is further increased by having had recent surgery [22]. Communication between medical tourists and health care professionals abroad may be difficult if there is a language barrier, thus limiting risk-related communication [23]. The quality of a hospital or of a physician's training may be difficult to ascertain if the credentials are foreign to the patient, further complicating patient understanding of risk [24]. In the case of a physician's error or hospital negligence, it is often difficult to receive compensation for malpractice in many countries [25]. Follow up care for medical tourists is uniquely challenging as they will be returning to their home country and may have difficulty contacting the physician or surgical team abroad should complications or questions arise post-operatively, which can pose as a post-operative health risk [14]. These potential risk factors have been previously identified, and many of them are preventable.

## Methods

To identify English-language Canadian medical tourism brokerage websites for inclusion in this analysis, we first compiled a listing of company names mentioned in published media accounts of medical tourism and reviewed for Canadian membership in the Medical Tourism Association (MTA). Following this we searched the identified company names in an internet search engine to locate their websites. Next we ran a series searches on two internet search engines, exhaustively searching combinations of the keywords Canad*, medical, tour*, and surger* using Boolean operators. Twenty two brokerages were initially identified using these strategies in summer of 2010. In reviewing their websites, five were excluded from the study as they appeared to be inactive (e.g., no current contact information), not focused on medical tourism (e.g., were selling insurance packages), or were not based in or operating out of Canada.

Following identification of the websites for inclusion in this analysis we performed a thematic content analysis. Thematic content analysis involves generating frequency counts of the dominant themes in a dataset that can be used to guide a thematic approach to analysis, which typically involves identifying themes within a dataset and comparing those themes to the study purpose and existing literature [26]. In keeping with this approach, the websites were first reviewed in order to develop a list of informational points that were commonly included. Additional informational points were added to the listing based upon our focus on risk communication and informed consent. Frequency counts of these informational points were generated, which formed the basis of the content component of our analysis. In reviewing the trends among the quantitative counts and comparing these trends to the existing literatures on medical tourism and informed consent, three themes were identified for the thematic component of the analysis. Following identification of these themes, consensus was sought among the team members to ensure confirmation of this interpretation. Data relevant to each of these themes were then compiled from the websites included in the study and reviewed again in order to focus the analysis.

## Results

A summary of the websites included in this analysis can be found in Table 1. A summary of the counts generated for the thematic content analysis is included in Table 2. In reviewing the content of the 17 websites included in this analysis, we found that Canadian medical tourism broker websites varied widely in scope, content, professionalism, and depth of information. Three themes emerged from the thematic content analysis: training and accreditation, risk communication, and business dimensions. In this section we examine each separately, identifying commonalities and differences across the reviewed websites. We provide direct quotations from the reviewed websites to illustrate the points raised in this section, and provide a number beside each quote (e.g., #2) that corresponds to 'company' column in Table 1.

**Table 1:** Medical Tourism Websites Included in Content Analysis

Company	Website	Province of Headquarters
1	http://timelymedical.ca	British Columbia

2	http://www.meditours.org	British Columbia

3	http://www.hsi-ssi.com/en	Quebec

4	http://www.surgicaltourism.ca	British Columbia

5	http://ecumedical.com/ecom.asp?*	Ontario

6	www.healthvacations.ca	None noted

7	http://www.debsonmedicaltourism.com	Quebec

8	http://www.medextra.com	Quebec

9	www.gosculptura.com	Quebec

10	http://ihcproviders.com	Ontario

11	http://passportmedical.com	British Columbia

12	http://www.intermedglobal.com	None noted

13	http://www.medipassionhealing.com*	Quebec

14	http://www.overseasmedicalservices.com*	Alberta

15	http://www.surgicalescape.com	Alberta

16	http://www.choicemedicalservices.com/	Manitoba

17	http://www.medasia.ca/	None noted

*Website used for study has either changed or been shut down since September 2010

**Table 2:** Informational points contained on Canadian medical tourism brokers' websites (N = 17)

Informational Points	number of websites including informational point	number of websites not including informational point
Evidence of professional membership or certification	6	11

Information on foreign physicians (e.g., certifications, training)	5	12

Explicit reference to foreign hospital accreditations	9	8

Explicit reference to foreign hospitals with JCI accreditation	6	11

Explicit reference to "medical tourism"	12	5

Explicit reference to "broker"	0	17

Explicit reference to "facilitator"	7	10

Tourism services offered (e.g., arranging sightseeing)	10	7

Images of tourist attractions	7	10

Health risks (general - e.g., surgical risks)	9	8

Health risks (specific to medical tourism - e.g., post-operative flight)	2	15

Patient testimonials	11	6

Pricing details	6	11

### Training & Accreditation

Three types of accreditation and/or training were mentioned explicitly on brokers' websites: (1) accreditation of international hospitals; (2) training and accreditation of international physicians; and (3) brokers' own training and accreditation. There was a certain degree of continuity between the three different types of training and accreditation identified; 29.4% of websites did not mention any specific credentials for the hospitals or physicians they referred to nor did they display logos of membership for their own brokerage. The remaining 70.6% varied in their level of professional accreditation and communication of physicians' qualifications.

The current gold standard for international hospitals is Joint Commission International (JCI) accreditation [14], and four of the websites stated specifically that they only work with hospitals that are JCI accredited, with one explaining: "JCI Accredited hospitals and clinics must have the same or exceed standards compared to the United States in clinical outcomes, infection rates, mortality rates and cleanliness." The majority of websites made no mention of any type of specific hospital accreditation or certification, often using vague descriptions as a substitute: "all rooms are equipped with the very latest American and European technology" (#9); "Our standards are as high, and can exceed, those in the United States and United Kingdom" (#13); the hospitals use the "best technology available on the market today" (#2). These examples show the language that was commonly used to advertise services, with superlative descriptors of the quality of service and availability of new technologies appearing on all websites.

In addition to indicators of the quality and accreditation of foreign medical facilities, websites provided information on the quality of the personnel in these facilities through reference to their medical credentials. Medical credentials of foreign physicians were mentioned on 41.2% of websites, sometimes in a generalized way: "many of our doctors have been trained in the United States and the United Kingdom, right alongside the physicians and surgeons that are working in the city in which you live" (#13). Overall 52.9% of websites stated that the company worked with international physicians trained, at least in part, in either North America or Europe. Some websites not reporting credentials and training used other tactics to gain the trust of potential medical tourists: "The life expectancy in Cuba is equal to that found in developed countries... This type of statistic can only be achieved if the medical training and procedures are similar to those of developed countries" (#16).

While there was no single, dominant professional organization or association to which the brokers belonged, 35.3% of the websites featured symbols and logos of some type on their main page, usually representing membership in various organizations (e.g., MTA, Better Business Bureau, International Medical Travel Association, Medical Travel and Health Quality Alliance). Demonstrations of certification, rather than simply membership, on the websites were rare, with only one website displaying the logo of the MTA certification for brokers. It is worth noting that the MTA membership logo is extremely similar to the certification logo (the caduceus) with the exception of the word "member" instead of "certification," a distinction which may not be apparent or have meaning to website visitors. One other site included a symbol for an accrediting agency, the Medical Travel and Health Tourism Quality Alliance, which certifies brokers to be either a Certified Medical Concierge or a Certified International Patient Manager.

### Risk Communication

A detailed search for any mention of risk on the reviewed websites revealed that 47.1% made no general or specific mention of risks at all. Of the 9 websites that did address risk, only two mentioned any specific risks associated with medical tourism, and these risks were mentioned only in order to illustrate how, by using a broker, they would be eliminated. Two sites mentioned that there was a small risk involved in obtaining a medical procedure, but were careful to separate that risk from any risks associated with traveling by reminding the patient that the same risk was involved with the medical procedure if done in the home country.

Risk of DVT due to post-operative travel was mentioned on two websites, but only in a context that implied that neither company's patients would have an increased risk of DVT due to their short flights. For example, a brokerage that only arranges for medical tourism between Canada and the United States lists several risks of traveling to a country outside of North America for surgery, including DVT due to long-haul flights. Their website provides a good example of how careful wording was often used on when discussing risk: "In the unlikely event of a complication during surgery, which is unrelated to the reason for traveling to the United States for medical care, the hospital will stabilize the patient and send him/her back to Canada by ambulance" (#1). Another example of careful wording is: "As with any other surgical procedure, there are risks and complications involved which are always measured. Certain risks depend on the individual surgeon and are avoided without being acknowledged due to the surgeon's expertise" (#9).

Discussion of negative outcomes and risks occurred most often on the Frequently Asked Question or Disclaimer sections of websites, and only 17.6% addressed possible negative outcomes. Such discussions appeared to be aimed at reassuring potential clients, as shown in this example: "As with any medical procedure at home or away, there is always a small chance of a complication, so one should always plan for this eventuality. That is why medical complication insurance is available...so that you can have peace of mind" (#11). While most websites provided minimal overviews of the possible negative outcomes to medical tourism, the majority had many examples of positive outcomes. One way this was done was through the inclusion of patient testimonials, where 70.6% of the websites contained at least one personal statement from a former patient or from a physician abroad. One company promised to put interested clients personally in touch with former clients for a live testimonial experience.

### Business Dimensions

Not surprisingly, websites conveyed information about the broker's business to potential medical tourists. One of the business dimensions covered pertained to brokers' involvement in follow up care, with 52.9% of the websites promising quality follow-up care upon return home. While those that did not make this promise typically did not address follow up care at all, one explicitly stated that the brokerage had no responsibility in overseeing such care, stating that: "once arrangements have been made to the mutual satisfaction of our client and their physician, we have no ongoing involvement in the doctor-patient relationship" (#1). For the brokers who were involved in arranging for it, follow up care services varied between companies. These services included arranging phone calls between the patient and the specialist abroad, having a report sent to the home physician, organizing rehabilitation, telehealth consultations, and answering questions. It was often unclear whether these services would come at an additional cost to the patient.

Medical tourism is often associated with price savings for patients. Nonetheless, only 29.4% of the websites included specific prices for various treatments. When sites did not list their prices, they often included percentages representing the average savings a tourist could expect. For example, one website stated that "if you have received quotes in America for $10,000, you can expect the same procedure to cost between $2,500 and $5,000" (#13) while another noted that "care comes at a cost that is 40-80% lower than the cost of the same procedures in the United States" (#6). It is interesting to note that United States dollars were quoted in these examples despite the fact that these companies were operating in Canada, although references to faster wait times for procedures typically quoted Canadian averages. For example, a website listed their services as being "4-10 times faster than in Canada" (#6).

The large majority (70.6%) of the brokers branded themselves as being involved in the business of 'medical tourism' via their websites. Brokers that did not use the medical tourism label instead used labels such as: medical travel, healthcare management, private pay medical services, and travel for health services. This divide in branding or labelling extended to the images used on sites, where medical tourism companies commonly depicted images of tropical flowers and palm trees, images of celebrations and festivals that occur in destination countries, and pictures of beach and ocean views, while sites not adopting the medical tourism label had little or no tourism-related imagery and were thus not marketing the tourism dimensions of international patient travel.

The term 'broker' or 'brokerage' was not used at all in the sample of reviewed websites. Meanwhile, this term is widely used in the academic and popular literature regarding medical tourism when characterizing these businesses and the services they offer [27-29]. The term that appeared most frequently (47.1%) was 'facilitator', and it appeared in a variety of permutations, including 'medical travel facilitator', 'health-medical tourism facilitator', and 'medical tourism facilitator.' Other descriptors included 'medical tourism service provider', 'referral agency', 'medical tourism advocates', 'international patient support' and 'medical tourism guidance.'

## Discussion

Medical tourism brokers play an important role in the medical tourism industry and have helped make traveling abroad for medical care a viable option for more people than ever before [6,7,11,12,14]. Through our assessment of 17 Canadian brokers' websites we have been able to gain an understanding of the quantity and quality of online information available to persons using Canadian brokers. Their websites were clearly designed to sell brokers' services to potential medical tourists. For this reason, the information provided through these websites is geared more toward promotional ends rather than helping to inform patients of the full range of their treatment options or communicating the potential risks inherent in medical tourism. Mason and Wright identified a similar trend in their analysis of 66 international medical tourism websites (which included broker and non-broker sites) [12]. Crooks et al.'s thematic content analysis of promotional brochures from medical tourism hospitals in India also revealed a strong emphasis on characterizing credentials and accreditation, services, and specializations over issues such as cost and follow up care [30]. When the findings of our analysis are considered in relation to these other studies it becomes clear that a lack of emphasis on risk communication in medical tourism marketing materials is not specific to Canadian brokers but, instead, is more likely an industry-wide phenomenon.

The findings shared above highlight many issues that hold significant implications for Canadian patients' informed consent when engaging in medical tourism, four of which we expand upon in this section. First, our review revealed that only 2 of the 17 brokerages had been certified by an accrediting body, while many websites had various logos signifying membership with the MTA and other groups. While these logos are meant to assure the patient of the broker's professional status, they do not typically guarantee any particular standard of operation. The risk generated by this lack of standardization is that it allows brokers with lower standards and who do less to mitigate and inform patients of risks to operate amidst competent brokers, with little means of differentiating between the two [6,14]. This lack of clarity may compromise a patient's ability to make informed decisions regarding their treatment, thereby ultimately compromising informed consent, and puts them in a position where they may be unsure of the consequences of deciding to use a particular broker to book a procedure abroad.

A second issue related to informed consent emerging from the findings pertains to the role of terminology in clarifying what services patients are purchasing. More specifically, the terminology chosen by the brokers may factor into how a patient views their services. The term 'medical tourism' has been described as that which is most commonly used within the industry and by the media [31], and the majority of the brokers in this study adopted it on their websites. Bioethicists and other scholars have observed that the term itself may be problematic; the word 'tourism' evokes images of beaches and sightseeing, potentially undermining the seriousness of the medical procedure in the patient's mind [19,31,32]. As such, the term may under-represent the risks involved in going abroad for medical care. Related to this is a need to develop standardized role terminology. The websites we reviewed uniformly avoided the term 'broker' that is so oft used in popularized contexts. This is likely deliberate; the term 'facilitator' evokes the image of a helper, someone making life easier, while the term 'broker' brings to mind the world of business and money-making. Moreover, a 'facilitator' may be seen more to have the patient's interests at heart, thus reducing risks, while a 'broker' might be seen as engaging in more mercenary, risk-taking behaviour, making the broker's perceived role an important element in the communication and understanding of risk.

The messages conveyed by the Canadian medical tourism broker websites we reviewed were overwhelmingly positive, which raises a third issue regarding informed consent. Positive, if not glowing, patient testimonials were more frequently present than not, while acknowledgement of risks were notably rare. This finding is consistent with those of other studies that have examined promotional information in medical tourism and have ultimately questioned the trustworthiness of what is being reported and its ability to support patient decision-making [7,14,30]. While it is understandable that medical tourism brokers, in an effort to portray their product in the most favourable light possible, may be reluctant to address the issue of risk on their websites, this omission is potentially misleading and unethical. Brokers may be inclined to leave the discussion of risk until later in the process of arranging care abroad or until they meet with the patient in person, but the website is likely the first and possibly main source of information for many patients [7]. In order to make a truly informed decision about whether or not to proceed with arrangements - and thus ultimately to give informed consent for medical care abroad - they ought to have access to balanced and accurate risk information at the beginning of their decision making process.

The final issue related to informed consent emerging from the findings of our thematic content analysis relates to information, or the lack thereof, about follow up care on the reviewed websites. Generally, follow up care details were rarely included in brokers' websites. This is concerning because when Canadians undergo surgery domestically, follow up care is commonly coordinated between the hospital and the family physician and is generally organized on behalf of the patient. Medical tourism disrupts this standard practice [33,34]. Medical tourists will inevitably be moving between two different health care systems that are not connected, making it unclear exactly who should be responsible for organizing check-ups and rehabilitation after the procedure [6]. If follow up care details are not shared with the patient initially, or if it is not noted explicitly that patients are expected to arrange for their own follow up care upon return home, they may decide to proceed with purchasing medical care abroad while being unaware of the amount of care needed once they have returned home. This poses a risk to medical tourists on many fronts. It may negatively impact their health should they not be in a position to navigate their home health care system upon return home without the support or referral of a domestic surgeon [1]. It may also lessen the quality of care patients receive upon returning home in that they may underestimate the informational needs of service providers thereby compromising care quality. One way to address these concerns is to encourage patients to communicate their plans to engage in medical tourism to their family doctors before going abroad as this may lead to consideration of follow up care before departure [30]. Such patients will have a broader range of sources from which they are gathering information and will be able to come to a more informed decision on whether or not they want to consent to the procedure.

As we have noted, ethical and legal debates about the regulation of the medical tourism industry exist in the scholarly literature [14,35]. In our own research we have found that Canadian medical tourism brokers believe they have significant roles to play not only within the industry but to medical tourists specifically, particularly to ensure these patients' safety [6]. The provision of informed consent falls naturally within the roles they espouse toward patients, but brokers' perspectives on this needs to be sought in order to ascertain whether or not they agree in both principle and practice.

The Canadian government is involved in protecting Canadian patients and ensuring their health through the public health care system, and as such is well positioned to address some of the issues that hold implications for medical tourists' informed consent that we have identified in this analysis. As brokers are involved in patient care, directly or indirectly, and have a unique relationship with the medical tourist, it would seem fitting that the government take steps to monitor their work and ensure they are meeting standards of care for patients. Turner has made a broad call for regulatory involvement by the American government in regard to medical tourism brokerage companies operating in the United States [14]. While the insights gained from our study of broker websites does not support the full range of regulations suggested by Turner, some of which would not protect patient choice (e.g., restricting brokers from advertising services not approved in their local jurisdictions), certain areas of intervention are justified by our findings. More specifically, Canadian government intervention in the areas of medical tourism brokers' involvement in follow up care and risk communication seems warranted given the lack of apparent consideration given to these domains by brokers currently as assessed by our review of their websites. Such intervention need not be complex, and could include developing informational resources for Canadian medical tourism brokers or requiring brokers to disclose risk information on their websites. In general, government regulators have an obligation to oversee and set standards for the information available to potential medical tourists in order to ensure that the risks of this practice are understood and that false, misleading, or unverifiable claims are not made by brokers that would unethically undermine patient informed consent [36].

## Limitations

One limitation is that the information we used to draw conclusions about brokers' business practices was limited to that which was found on their websites. We are aware that many brokers use brochures, information booklets, e-mails, and face-to-face conversations in addition to their websites as means of disseminating information to their clients; these other forms of communication may provide valuable information not found on the websites. That being said, we do believe that the websites are a key source of information and that they play a significant role in patients' decision-making processes and formation of initial impressions when considering medical tourism and so warrant explicit consideration.

Another limitation is that the sample of websites reviewed may not be exhaustive of Canada-based brokers. We made all reasonable efforts to identify medical tourism brokerages based in Canada with English-language websites; however, it is possible that there are more companies currently operating in Canada than were identified in this study (e.g., those operating exclusively in French). Additionally, we did not include companies whose primary business was standard travel and tourism but who also had a medical tourism component. Nonetheless, we believe the included websites serve as a representative sample of medical tourism brokers in Canada and certainly those most accessible to potential medical tourists online. It is worth noting that three of the websites reviewed have been subsequently shut down or are under construction, an issue similarly encountered by Cormany and Baloglu in their analysis of international medical travel websites [29]. Our analysis, therefore, is of a representative snapshot of an evolving industry.

## Conclusions

Our review of 17 Canadian medical tourism broker websites revealed that medical tourists may be exposed to incomplete or misleading information about the risks involved with this practice. Training and accreditation of these brokers was uneven and the symbols of professionalization found on these websites were more likely to confuse than enlighten potential medical tourists. The terminology used by brokers may obfuscate their roles, including the place of tourism activities in medical tourism. Broker websites were also found generally not to provide information on the risks of this practice and information on the brokers' business, including pricing and the provision of follow up care. These shortcomings are matters of grave ethical concern as they threaten the ability of patients to make informed decisions regarding their medical care.

Our findings support increased government oversight of brokers in the name of improving the communication of risks to patients and better informing their choice regarding whether or not to engage in medical tourism, thereby ultimately enhancing informed consent. Protecting patient choice justifies some previously called-for interventions, including government review of broker accreditors, oversight of the information provided on websites, and requirements for transparency regarding the provision of follow up care and legal liability [14,27]. Other regulations are not justified by an interest in informed consent and may undermine patient choice. Creating support for the development of oversight mechanisms for the medical tourism industry will be aided by continued study of medical tourism brokers, including other country-specific studies like our own and reviews of the information provided by brokers beyond their publicly accessible websites.

## Abbreviations

DVT: deep-vein thrombosis; JCI: Joint Commission International; MTA: Medical Tourism Association.

## Competing interests

The authors declare that they have no competing interests.

## Authors' contributions

KP wrote the background, results, discussion, and conclusion sections and gathered website data for this analysis. JS contributed to the drafting and editing of the background, results, discussion, and conclusion sections and to data analysis. VC wrote the methods section, edited the full manuscript, and contributed to data analysis. RJ aided in identifying the list of brokers, edited the full manuscript, and contributed to data analysis. All authors read and approved the final manuscript.

## Pre-publication history

The pre-publication history for this paper can be accessed here:

http://www.biomedcentral.com/1472-6939/12/17/prepub

## References

[B1] Crooks VA Kingsbury P Snyder J Johnston R What is known about the patient's experience of medical tourism? a scoping review BMC Health Serv Res 2010 10 266 10.1186/1472-6963-10-266 20825667 PMC2944273

[B2] Abdullah BJJ The sky is falling Biomed Imaging Intervention J 2006 2 e29 10.2349/biij.2.3.e29 PMC3097626 21614242

[B3] Connell J Medical tourism: sea, sun, sand and... surgery Tourism Manage 2006 27 1093 1100 10.1016/j.tourman.2005.11.005

[B4] Morrow A Man dies after controversial MS treatment, doctor says http://www.theglobeandmail.com/news/national/man-dies-after-controversial-ms-treatment-doctor-says/article1805379/

[B5] Jacobs M Fixing foreign foul-ups http://www.edmontonsun.com/comment/columnists/mindelle_jacobs/2010/12/07/16465056.html

[B6] Snyder J Crooks VA Kingsbury P Adams K Johnston R The 'Patient's Physician One-Step Removed': The evolving roles of medical tourism facilitators J Med Ethics 2011 37 Suppl 9 530 534 21478420 10.1136/jme.2011.042374

[B7] Lunt N Hardey M Mannion R Nip, tuck and click: medical tourism and the emergence of web-based health information Open Med Info J 2010 4 1 11 10.2174/1874431101004010001 PMC2874214 20517465

[B8] Turner L "Medical tourism" and the global marketplace in health services: U.S. patients, international hospitals, and the search for affordable health care Int J Health Serv 2010 40 443 67 10.2190/HS.40.3.d 20799670

[B9] Klaus M Outsourcing vital operations: What if US health care costs drive patients overseas for surgery? Quinnipiac Health Law J 2006 9 219 247

[B10] Impicciatore P Pandolfini C Bonati M Reliability of health information for the public on the world wide web: systematic survey of advice on managing fever in children at home BMJ 1997 314 1875 1879 9224132 10.1136/bmj.314.7098.1875 PMC2126984

[B11] Cormany D Baloglu S Medical travel facilitator websites: an exploratory study of web page contents and services offered to the prospective medical tourist Tourism Manage 2010

[B12] Mason A Wright KB Framing medical tourism: An examination of appeal, risk, convalescence, accreditation, and interactivity in medical tourism websites J Health Comm 2011 16 Suppl 2 1 15 10.1080/10810730.2010.535105 21161812

[B13] Beauchamp TL Childress JF Principles of Biomedical Ethics 2001 5 New York: Oxford University Press

[B14] Turner L Quality in health care and globalization of health services: accreditation and regulatory oversight of medical tourism companies Int J Qual Health Care 2011 23 1 7 10.1093/intqhc/mzq078 21148210

[B15] Manson NC O'Neill O Rethinking informed consent in bioethics 2007 New York: Cambridge University Press

[B16] Paling J Strategies to help patients understand risks BMJ 2003 327 745 14512489 10.1136/bmj.327.7417.745 PMC200818

[B17] Ball LK Evans G Bostrom A Risky business: challenges in vaccine risk communication Pediatrics 1998 101 453 458 10.1542/peds.101.3.453 9481013

[B18] Bogardus ST Jr Holmboe E Jekel JF Perils, pitfalls, and possibilities in talking about medical risk JAMA 1999 281 1037 1041 10.1001/jama.281.11.1037 10086441

[B19] Kangas B Hope from abroad in the international medical travel of Yemeni patients Anthropol Med 2007 14 293 305 10.1080/13648470701612646 27268744

[B20] MacReady N Developing countries court medical tourists Lancet 2007 369 1849 1850 10.1016/S0140-6736(07)60833-2 17549816

[B21] Ansari MT Cheung MY Huang JQ Eklof B Karlberg JPE Traveler's thrombosis: a systematic review J Travel Med 2005 12 142 145 15996443 10.2310/7060.2005.12303

[B22] Page SJ The evolution of travel medicine: a new research agenda for tourism? Tourism Manage 2009 30 149 157 10.1016/j.tourman.2008.04.011 PMC7115677 32287728

[B23] Reed CM Medical tourism Med Clin NA 2008 92 1433 1446 10.1016/j.mcna.2008.08.001 PMC7127449 19061760

[B24] Howze KS Medical tourism: symptom or cure? Ga L Rev 2007 41 1013 1050

[B25] Terry NP Under-regulated health care phenomena in a flat world: medical tourism and outsourcing West New Eng L Rev 2007 29 421 72

[B26] Weber RP Basic Content Analysis 1990 2 Newbury Park, CA: Sage

[B27] Spece RG Jr Medical tourism: protecting patients from conflicts of interest in broker's fees paid by foreign providers Ariz Legal Stud: Discussion Paper No. 09-41 2009

[B28] Carrera P Lunt N A European perspective on medical tourism: the need for a knowledge base Int J Health Serv 2010 40 469 84 10.2190/HS.40.3.e 20799671

[B29] Burkett L Medical tourism: concerns, benefits, and the American legal perspective J Legal Med 2007 28 223 45 10.1080/01947640701357763 17558794

[B30] Crooks VA Turner L Snyder J Johnston R Kingsbury P Promoting medical tourism to India: messages, images, and the marketing of international patient travel Soc Sci Med 2011 72 726 732 10.1016/j.socscimed.2010.12.022 21310519

[B31] Turner L 'First world health care at third world prices': globalization, bioethics and medical tourism BioSocieties 2007 2 303 25 10.1017/S1745855207005765

[B32] Johnston R Crooks VA Snyder J Kingsbury P What is known about the effects of medical tourism in destination and departure countries? a scoping review Int J Equity Health 2010 9 24 10.1186/1475-9276-9-24 21047433 PMC2987953

[B33] Turner L Medical tourism: family medicine and international health-related travel Can Fam Physician 2007 53 1639 1641 17934018 PMC2231416

[B34] Crooks VA Snyder J Medical tourism: what Canadian family physicians need to know Can Fam Physician 2011 57 Suppl 5 527 529 21571709 PMC3093576

[B35] Crooks VA Snyder J Regulating medical tourism Lancet 2010 376 Suppl 9751 1465 1466 21036273 10.1016/S0140-6736(10)61993-9

[B36] Faden RR Beauchamp TL King NMP A history and theory of informed consent 1986 New York: Oxford University Press 11621442

